# Predictive Model and Precaution for Oral Mucositis During Chemo-Radiotherapy in Nasopharyngeal Carcinoma Patients

**DOI:** 10.3389/fonc.2020.596822

**Published:** 2020-11-05

**Authors:** Pei-Jing Li, Kai-Xin Li, Ting Jin, Hua-Ming Lin, Jia-Ben Fang, Shuang-Yan Yang, Wei Shen, Jia Chen, Jiang Zhang, Xiao-Zhong Chen, Ming Chen, Yuan-Yuan Chen

**Affiliations:** ^1^Department of Radiation Oncology, Cancer Hospital of University of Chinese Academy of Sciences (Zhejiang Cancer Hospital), Institute of Cancer and Basic Medicine (IBMC), Chinese Academy of Sciences, Zhejiang Key Laboratory of Radiation Oncology, Hangzhou, China; ^2^Department of Radiation Oncology, Quanzhou First Hospital Affiliated to Fujian Medical University, Quanzhou, China; ^3^First Tumor Department, People’s Hospital of Maoming, Maoming, China; ^4^Radiation Center, Shanghai Pulmonary Hospital, Shanghai, China; ^5^AI Research Institute, Hangzhou YITU Healthcare Technology Co. Ltd., Hangzhou, China

**Keywords:** nasopharyngeal carcinoma, radiotherapy, radiation-induced oral mucositis, dosimetric parameter, preventive measures

## Abstract

**Purpose:**

To explore risk factors for severe acute oral mucositis of nasopharyngeal carcinoma (NPC) patients receiving chemo-radiotherapy, build predictive models and determine preventive measures.

**Methods and Materials:**

Two hundred and seventy NPC patients receiving radical chemo-radiotherapy were included. Oral mucosa structure was contoured by oral cavity contour (OCC) and mucosa surface contour (MSC) methods. Oral mucositis during treatment was prospectively evaluated and divided into severe mucositis group (grade ≥ 3) and non-severe mucositis group (grade < 3) according to RTOG Acute Reaction Scoring System. Nineteen clinical features and nineteen dosimetric parameters were included in analysis, least absolute shrinkage and selection operator (LASSO) logistic regression model was used to construct a risk score (RS) system.

**Results:**

Two predictive models were built based on the two delineation methods. MSC based model is more simplified one, it includes body mass index (BMI) classification before radiation, retropharyngeal lymph node (RLN) area irradiation status and MSC V55%, RS = −1.480 + (0.021 × BMI classification before RT) + (0.126 × RLN irradiation) + (0.052 × MSC V55%). The cut-off of MSC based RS is −1.011, with an area under curve (AUC) of 0.737 (95%CI: 0.672-0.801), a specificity of 0.595 and a sensitivity of 0.786. OCC based model involved more variables, RS= −4.805+ (0.152 × BMI classification before RT) + (0.080 × RT Technique) + (0.097 × Concurrent Nimotuzumab) + (0.163 × RLN irradiation) + (0.028 × OCC V15%) + (0.120 × OCC V60%). The cut-off of OCC based RS is −0.950, with an AUC of 0.767 (95%CI: 0.702–0.831), a specificity of 0.602 and a sensitivity of 0.819. Analysis in testing set shown higher AUC of MSC based model than that of OCC based model (AUC: 0.782 vs 0.553). Analysis in entire set shown AUC in these two method-based models were close (AUC: 0.744 vs 0.717).

**Conclusion:**

We constructed two risk score predictive models for severe oral mucositis based on clinical features and dosimetric parameters of nasopharyngeal carcinoma patients receiving chemo-radiotherapy. These models might help to discriminate high risk population in clinical practice that susceptible to severe oral mucositis and individualize treatment plan to prevent it.

## Introduction

Nasopharyngeal carcinoma (NPC) has an extremely uneven endemic distribution within Southern China and Southeast Asia (1). The mainstay treatment for this disease is chemo-radiotherapy. Both traditional intensity-modulated radiation therapy (IMRT) including volumetric-modulated arc therapy and fixed-field intensity-modulated radiotherapy and advanced IMRT technique like helical tomography radiotherapy (TOMO) are commonly used in the treatment of NPC. Almost all NPC patients receiving chemo-radiotherapy will develop into a certain degree of acute oral mucositis during treatment. Morbidity of severely acute oral mucositis is 20%–40% (2–4). Severe oral mucositis causes pain, reduces oral intake, impairs quality of life, affects treatment compliance, gives raise to secondary infection, all of which lead to increase treatment cost and might impact prognosis of the disease (5–9). Moreover, studies reported a correlation between severity of acute and late reaction, severe acute reactions implicated in the subsequent development of late radiation toxicity (10–12). Currently, available medicine for prevention and treatment of oral mucositis are not effective enough according to MASCC/ISOO clinical practice guidelines (13).

Dose-dependent is one of the most important features for the morbidity of severe oral mucositis in NPC patients receiving radiotherapy (14–17). Previous studies evaluated predictive effect for severe oral mucositis of dosimetric parameters by using two oral mucosa structure contour methods (oral cavity contour, OCC and mucosa surface contour, MSC) and identified OCC V30% and MSC V50% as predictive factors in NPC patients receiving traditional IMRT (2, 18). However, other clinical features were not taken into account in these two studies. Considering heterogeneity among patients, we assumed that incidence and severity of oral mucositis were influenced by factors such as individual sensitivity, disease severity and treatment scheme as well as irradiation dose. Therefore, it’s important to identify susceptible factors of severe oral mucositis and to intervene them in advance. Whereas, there is a scarcity of effective models to predict risk of severe oral mucositis in NPC patients receiving chemo-radiotherapy. This lack of knowledge limits the ability to identify patients at risk of severe oral mucositis and explore effective prevention measures. In this study, we explore risk factors for severely acute oral mucositis in NPC patients receiving chemo-radiotherapy, build predictive models and determine potential measures, by which clinicians can find a good way to prevent severe oral mucositis and to easily communicate with patients.

## Methods and Materials

### Patient Eligibility and Data Collection

A total of 270 newly diagnosed NPC patients treated from November 2016 to June 2019 were included in this study. Clinic data such as age, gender, comorbidity, smoking/drinking status, treatment information, severity of mucositis etc. were collected. Absolute cumulative dose-volume of interesting structures as dosimetric parameter (volume, mean dose (D_mean_), maximum dose (D_max_), median dose (D_med_), minimum dose (D_min_) and V5%–V70% in 5Gy interval) were exported from RayStation V3.0 system. Vx% means volume percentage of structure receiving dose ≥ x Gy.

### Treatment

#### Chemotherapy and Target Treatment

All patients received 0–4 cycle(s) of platinum-based induction chemotherapy followed by radical radiotherapy plus 0–3 cycle(s) of concurrent chemotherapy (All patients had received at least one cycle of chemotherapy). Concurrent chemotherapy was prescribed as: (i) cisplatin 80–100 mg/m^2^ on day 1, every 3 weeks; (ii) nedaplatin 80–100/mg/m^2^ on day 1, every 3 weeks; and (iii) carboplatin was dosed to the target area under the concentration-time curve of 5 on day 1, every 3 weeks; (iv) orally capecitabine, tegafur or S1 was used during radiotherapy when the mentioned three agents were unsuitable in a small part of patients (17, 6.3%). Concurrent nimotuzumab, a humanized anti-EGFR IgG1 monoclonal antibody, was given to a part of patients according to their intention, 200mg intravenously every week during radiation (19).

#### Radiotherapy

All patients in this study conducted radical radiation, 168 patients conducted TOMO radiotherapy and 102 patients received traditional IMRT. Mask fabrication, fixation of position and radiation plan has been reported in a previous study (2). Oral mucosa structure was contoured using both OCC and MSC methods. Two clinical oncologists performed and reviewed structure contouring. OCC method included region as recommended in a previous study (20): above to hard palate, underneath to floor of mouth, anterior to the buccal mucosa around the teeth, posterior to tongue surface and uvula. While MSC method defined the oral mucosa as a 3 mm thick wall of tissue based on research by Ueno et al. (21). It includes mucosa surface of buccal mucosa, buccal gingiva, gingiva proper, lingual gingiva, lingual frenulum, alveolar mucosa, labial mucosa, labial gingiva, labial frenulum, mucosal surface of the floor of the mouth, mucosal surface of tongue anterior to the terminal sulcus, mucosal surface of the hard palate, and the inferior mucosal surface of the soft palate.

#### Basic Oral Care

All patients in this study visited the dentist before radiation and dealt with the potential problem like decayed tooth etc. All of them received oral care education at the time of admission and performed basic oral care during radiotherapy, including routine checkup and cleaning (e.g. brushing teeth and rinsing mouth). Administration, such as amifostine (22) and recombinant human interleukin-11 oxygen atomization (23), was carried out in some patients from the beginning of radiation.

### Toxicity Assessment

Toxicity was consistently scored for all patients according to EORTC/RTOG criteria of acute effects for mucous membrane. Grade 0: no change over baseline, Grade 1: injection/may experience mild pain not requiring analgesia, Grade 2: patchy mucositis which may produce an inflammatory serosanguinitis discharge/may experience moderate pain requiring analgesia, Grade 3: confluent fibrinous mucositis/may include severe pain requiring narcotic, Grade 4: ulceration, hemorrhage or necrosis. Toxicities were prospectively and weekly recorded for patients prior to the start of radiation during radiation by oncologists trained in the use of the scoring systems. The toxicity endpoint of interest chosen for analysis was the maximum reported mucositis grade, dichotomized into severe oral mucositis group (maximum toxicity scored ≥ 3) and non-severe oral mucositis group (maximum toxicity score < 3). No patient was found with baseline toxicity of oral mucosa.

### Statistical Analysis

The mean value comparisons of continuous variables were performed by t-test. Chi-square test was performed in comparison of categorical variables. Nineteen clinic factors including age, gender, smoking, drinking, diabetes, hypertension, BMI before radiation, RT technique, total radiation time, T stage, N stage, clinic stage, concurrent chemotherapy, concurrent nimotuzumab, glycididazole sodium (GSI) during radiation, amifostine, interleukin-11 (IL-11) oxygen atomization, retropharyngeal lymph node (RLN) area irradiation, Ib area irradiation and nineteen dosimetric objectives including volume of structure, mean dose, median dose, maximum dose, minimum dose and V5%-V70% in an interval of 5Gy were involved in analysis. Two multivariate prediction models were independently trained from two sets of predictors (OCC based and MSC based). All patients split into training set and testing set by using cross-validation-based regularization factor selection. LASSO logistic regression was chosen to construct a risk score (RS) model. Receiver operating characteristic (ROC) curve analysis was conducted to evaluate the performance of predictive models, then determine optimal RS cut-off and dose restriction standard separating high and low risk for severe oral mucositis. All analyses were performed using R software (R version 4.0.2; Tableone, glmnet package, caret package, lattice package, pROC package, plyr package, ggplot2 package, foreach package and Matrix package).

## Results

Clinic characteristics were shown in **Table 1**. The median age was 50 years (range, 16–77 years). The male-female ratio was 3.3:1. Eighty-eight (32.6%) patients underwent grade 1 mucositis, 102 (37.8%) patients underwent grade 2 mucositis and 80 (29.6%) patients underwent grade 3 mucositis.

**Table 1:** Clinic characteristics of 270 nasopharyngeal carcinoma patients.

Characteristics		Mucositis		
		Grade 0-2 (N=190)	Grade 3 (N=80)	P value
Age (year) Mean (SD)		51.32 (11.06)	49.68 (10.98)	0.266
Gender (n, %)	Male	147(77.4)	60(75.0)	0.793
	Female	43(22.6)	20(25.0)	
Diabetes (n, %)	No	178(93.7)	73(91.2)	0.650
	Yes	12(6.3)	7(8.8)	
Hypertension (n, %)	No	137(72.1)	58(72.5)	1.000
	Yes	53(27.9)	22(27.5)	
Smoking (n, %)	No	111(58.4)	39(48.8)	0.185
	Yes	79(41.6)	41(51.2)	
Drinking (n, %)	No	131(68.9)	53(66.3)	0.771
	Yes	59(31.1)	27(33.7)	
BMI before RT (n, %)	underweight	3(1.5)	2(2.5)	0.022
	normal weight	90(47.4)	21(26.3)	
	overweight	49(25.8)	26(32.5)	
	obesity class I	43(22.6)	26(32.5)	
	obesity class II	5(2.6)	5(6.3)	
T stage (n, %)	T_1_	14 (7.4)	8 (10.0)	0.453
	T_2_	27 (14.2)	11 (13.8)	
	T_3_	92 (48.4)	31 (38.8)	
	T_4_	57 (30.0)	30 (37.5)	
N stage (n, %)	N_0_	9 (4.7)	2 (2.5)	0.290
	N_1_	74 (38.9)	24 (30.0)	
	N_2_	85 (44.7)	40 (50.0)	
	N_3_	22 (11.6)	14 (17.5)	
Clinic stage (n, %)	I	2 (1.1)	0 (0.0)	0.206
	II	18 (9.5)	5 (6.2)	
	III	97 (51.1)	34 (42.5)	
	IV	73 (38.4)	41 (51.2)	
C-Chemotherapy (n, %)	No	24(12.6)	10(12.5)	1.000
	Yes	166(87.4)	70(87.5)	
C-Nimotuzumab (n, %)	No	89(46.8)	27(33.8)	0.064
	Yes	101(53.2)	53(66.2)	
GSI (n, %)	No	41(21.6)	23(28.8)	0.268
	Yes	149(78.4)	57(71.2)	
Amifostine (n, %)	No	91(47.9)	36(45.0)	0.763
	Yes	99(52.1)	44(55.0)	
IL-11 (n, %)	No	87(45.8)	31(38.8)	0.352
	Yes	103(54.2)	49(61.2)	
RT technique (n, %)	Traditional IMRT	80(42.1)	22(27.5)	0.034
	TOMO	110(57.9)	58(72.5)	
RTT Mean (Q_25_, Q_75_)		45(44-47)	45(44-47)	0.748
RLN (n, %)	None	27(14.2)	3(3.8)	0.011
	Unilateral	55(28.9)	18(22.5)	
	Bilateral	108(56.8)	59(73.8)	
I b (n, %)	None	134(70.5)	51(63.8)	0.351
	Unilateral	46(24.2)	26(32.5)	
	Bilateral	10(5.3)	3(3.8)	

RT, radiation; BMI, body mass index; underweight = BMI before RT ≥16.5 kg/m^2^,<18.5kg/m^2^; normal weight = BMI before RT ≥18.5 kg/m^2^,<23.0kg/m^2^; overweight = BMI before RT≥23.0 kg/m^2^,<25.0kg/m^2^; obesity class I = BMI before RT≥25.0 kg/m^2^,<30.0kg/m^2^; obesity class II = BMI before RT≥30.0 kg/m^2^,<40.0kg/m^2^; C-Chemotherapy, concurrent chemotherapy; C-Nimotuzumab, concurrent nimotuzumab; GSI, glycididazole sodium for injection; IL-11, recombinant human interleukin-11; IMRT, intensity-modulated radiotherapy; TOMO, helical tomography radiotherapy; RTT, radiation total time; RLN, retropharyngeal lymph node region irradiation; I b, I b region irradiation; SD, standard deviation; M, median; Q, quartile.

### Relationship Between Dosimetric Objectives and Severe Oral Mucositis

**Figure 1:**
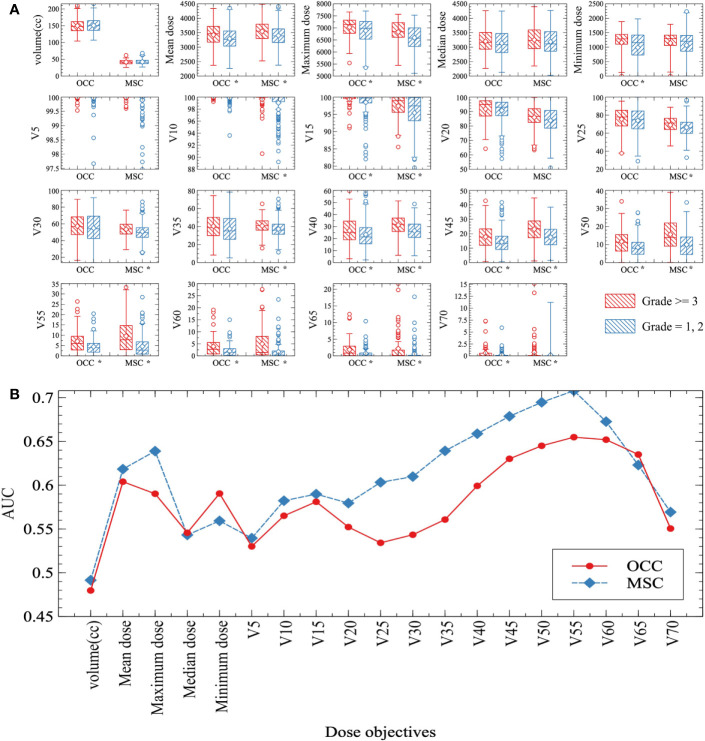
**(A)** Box plots of dose-volume objectives distributions. Mean values are indicated by the horizontal lines within boxes and median values are represented by the diamonds. Severe oral mucositis group (grade ≥ 3) and non-severe oral mucositis group (grade = 1, 2) data were drawn as red forward diagonal and blue backward diagonal boxes respectively. Note: *Statistically significant at p=0.05 level. **(B)** Area under curve (AUC) of all the dose-volume objectives under both oral cavity contour (OCC) (red solid line) and mucosa surface contour (MSC) (blue dashed line) methods. Each AUC is acquired from the ROC curve of each objective. Most dose-volume objectives under MSC method show better performance (higher AUC) than OCC method in terms of predicting severe oral mucositis.

The distribution of each dose-volume objectives from severe oral mucositis group (grade ≥ 3) and non-severe oral mucositis group (grade < 3) patients were compared as shown in **Figure 1A**. Distinctively smaller values of non-severe oral mucositis group patients can be directly observed for mean dose, maximum dose, minimum dose, V10%–V65% from plots. Most dosimetric parameters were significantly correlated with the occurrence of severe oral mucositis (P < 0.05). Then, we performed univariate ROC analysis for all dosimetric objectives. The predictive power was quantified as area under curve (AUC) which acquired from the ROC curve for each objective as shown in **Figure 1B**. Better performance can be observed in objectives under MSC method in general in terms of predicting severe oral mucositis and the highest AUC was seen in MSC V55%.

### RS Model for Severe Oral Mucositis

Age and all dosimetric parameters were analyzed as continuous variables, other clinic features were included as categorical variables. BMI before RT was divided into 7 levels according to WHO BMI cut-offs in Asian population (24): BMI <16.5 kg/m^2^ was severely underweight, BMI ≥16.5 kg/m^2^ and <18.5 kg/m^2^ was underweight, BMI≥18.5 kg/m^2^ and <23.0 kg/m^2^ was normal weight, BMI ≥23.0 kg/m^2^ and <25.0 kg/m^2^ was overweight, BMI ≥25.0 kg/m^2^ and <30.0 kg/m^2^ was obesity class I, BMI ≥30.0 kg/m^2^ and <40.0 kg/m^2^ was obesity class II and ≥40.0 kg/m^2^ was obesity class III. No severely underweight and obesity class III patients was found in this study. Irradiation status of RLN area and Ib lymph node area was divided into 3 levels: none irradiation, unilateral irradiation and bilateral irradiation. T stage, N stage and clinic stage were divided into 4 levels. Other variables were divided into two levels as shown in **Table 1**.

**Figure 2:**
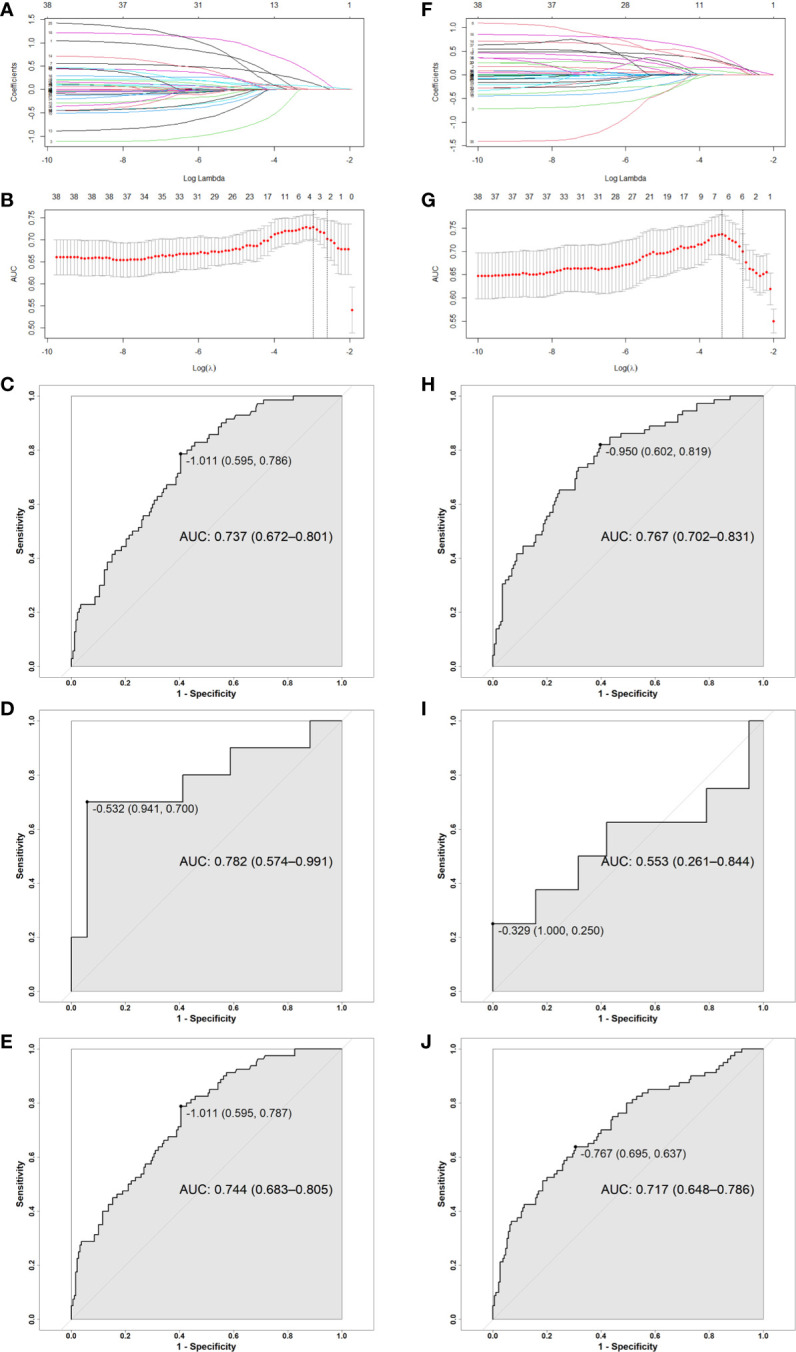
**(A**, **F)** LASSO coefficient profiles of 19 clinical features and 19 MSC and OCC based dosimetric parameters; **(B**, **G)** Ten-fold cross-validation for tuning parameter selection in MSC and OCC based LASSO model. **(C–E, H–J)** ROC curve for MSC and OCC based model: **(C, H)** Training group, **(D, I)** Testing group, **(E, J)** Entire group. The point on the curve is cutoff value for RS and the following bracket contains specificity and sensitivity. Abbreviation: RS, risk score; OCC, oral cavity contouring; MSC, mucosa surface contouring; AUC, area under curve; CI, confidence interval.

To construct MSC based and OCC based predictive models, we chose penalized LASSO regression model to calculate a RS by using above 38 features. LASSO coefficient profiles of 19 clinical features and 19 dosimetric objectives in each model were shown in **Figures 2A, F**. Ten-fold cross-validation was used to select an optimal model. We chose lambda.1se for model filtering, as is shown in **Figures 2B, G**. Finally, two predictive models were generated by training set. MSC based model involves less variables (2 clinical features and 1 dosimetric objective), the function is RS= −1.480 + (0.021 × BMI classification before RT) + (0.126 × RLN irradiation) + (0.052 × MSC V55%). The cut-off of MSC based RS is -1.011, with an area under curve (AUC) of 0.737 (95%CI: 0.672-0.801), a specificity of 0.595 and a sensitivity of 0.786. Function of OCC based model is RS= −4.805 + (0.152 × BMI classification before RT) + (0.080 × RT Technique) + (0.097 × Concurrent nimotuzumab) + (0.163 × RLN irradiation) + (0.028 × OCC V15%) + (0.120 × OCC V60%). The cut-off of OCC based RS is -0.950, with an AUC of 0.767 (95%CI: 0.702-0.831), a specificity of 0.602 and a sensitivity of 0.819. Analysis in testing set shown higher AUC of MSC based model than that of OCC based model (AUC: 0.782 vs 0.553). Analysis in entire set shown AUC in these two method-based models were close (AUC: 0.744 vs 0.717). As is shown in **Figures 2C–E, H–J**. Assignment of involved variables are as follows: BMI classification before RT (underweight = 1, normal weight= 2, overweight = 3, obesity class I = 4, obesity class II = 5), RLN (none = 0, unilateral = 1, bilateral = 2), RT technique (traditional IMRT = 1, TOMO = 2), concurrent nimotuzumab (no = 0, yes = 1), Vx% = volume percentage of OCC or MSC receiving dose ≥ x Gy.

### Dose Limitation for Vx% Involved in Models

MSC is a more accurate contouring method when compared with OCC. MSC based model involves only one dosimetric objective, V55%. Then, OCC based model involves two dosimetric objectives, V15% and V60% and these two objectives have to be limited at the same time. To make it easy for physicians to give a dose restriction in radiation plan, we performed ROC analysis for the mentioned dosimetric objectives. The cut-off of MSC V55% is 2.565%, with an AUC of 0.708, a sensitivity of 0.838 and a specificity of 0.484. The cut-off of OCC V15% is 99.523%, with an AUC of 0.582, a sensitivity of 0.762 and a specificity of 0.463. The cut-off of OCC V60% is 3.270%, with an AUC of 0.652, a sensitivity of 0.475 and a specificity of 0.816. ROC curves are shown in supplement data: **Figures S1–S3** (**Supplementary Data**).

### Effects of Chemotherapy Agent and Nimotuzumab on Radiation Mucositis

There was not significant correlation between the development of severe oral mucositis and chemotherapy in the regression equation. Considering radiation combined with concurrent chemotherapy is the standard treatment at present, chi-square test was run in chemotherapy subgroup to determine whether chemotherapy agent had impact on incidence of severe oral mucositis. As shown in **Table S1** (**Supplementary Data**), incidence of severe oral mucositis in patients receiving non-platinum chemotherapy (Capecitabine, Xeloda and S1) was significantly higher than that in patients receiving platinum concurrent chemotherapy (52.9% vs 27.9%, p=0.029).

Concurrent nimotuzumab was a small weighted risk factor in OCC based model. Subgroup analyses in patients with and without concurrent chemotherapy were conducted. There were 236 patients received concurrent chemotherapy in this study, of which 46 in 129 patients (35.7%) with concurrent nimotuzumab and 24 in 107 patients (22.4%) without concurrent nimotuzumab suffered ≥ grade 3 oral mucositis (p=0.027). While no significant correlation between concurrent nimotuzumab and incidence of severe oral mucositis was found in 34 patients without concurrent chemotherapy (p =0.763, as shown in **Table S2**, **Supplementary Data**).

### Effects of Radiation Techniques on Radiation Mucositis

**Figure 3:**
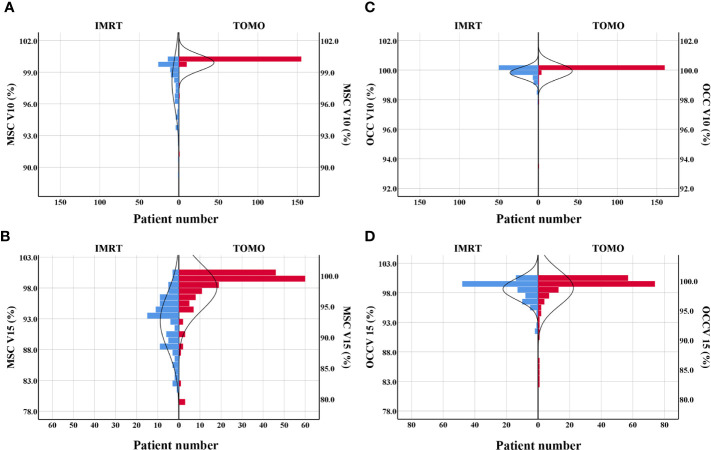
Patients distribution of V10% and V15% by using TOMO and IMRT. **(A)** MSC V10%, **(B)** MSC V15%, **(C)** OCC V10%, **(D)** OCC V15%. Abbreviations: OCC, oral cavity contouring; MSC, mucosa surface contouring.

This study indicated that TOMO was a risk factor for severe oral mucositis in OCC based model. A Mann-Whitney U test was run to determine if there were differences in dose-volume percentage between patients receiving traditional IMRT and TOMO. **Figures 3A–D** directly shown more patients had high level of V10%-V15% in TOMO group than in traditional IMRT group. Median value of V10%-V15% under OCC and MSC in TOMO was significantly higher than those in traditional IMRT, as shown in **Table S3** (**Supplementary Data**).

## Discussion

Clinically, patients received the same dose of radiotherapy and same intensity of chemotherapy sometimes undergo different degrees of oral mucositis. It might due to heterogeneity among individuals, disease features, treatment relevant factors. In the present study, we found the dose-volume percentage were strongly associated with occurrence of severe oral mucositis, other important factors include BMI classification before radiation, RT technique, RLN area irradiation status and application of concurrent nimotuzumab. Based on these factors, two risk score models were built.

OCC based model includes more variables than MSC based model, they are concurrent nimotuzumab and RT technique. OCC encompasses the whole oral cavity, which is a rougher way to evaluate dose distribution in oral cavity rather than oral mucosa. Thus, when applied in oral mucositis, it would be influenced by more factors. Nimotuzumab is an IgG1 humanized monoclonal antibody directed against the extracellular domain of the EGFR blocking the binding to its ligands. Several studies demonstrated that nimotuzumab combined with neo-adjuvant chemotherapy or concurrent chemotherapy brought overall survival benefit (25–27). OCC based model in this study shows that nimotuzumab will enhance the incidence of severe oral mucositis when it is along with concurrent chemotherapy. Nevertheless, the weight of this factor is relatively small in the function. The use of nimotuzumab is not opposed when radiation dose is strictly limited. However, the patient must be fully informed and emphasized with the importance of oral care during treatment. Recently, a randomized phase III non-inferiority study of radiotherapy plus concurrent nimotuzumab versus cisplatin in stage II-III NPC patients (NCT 03837808) and we are looking forward the results.

**Figure 4:**
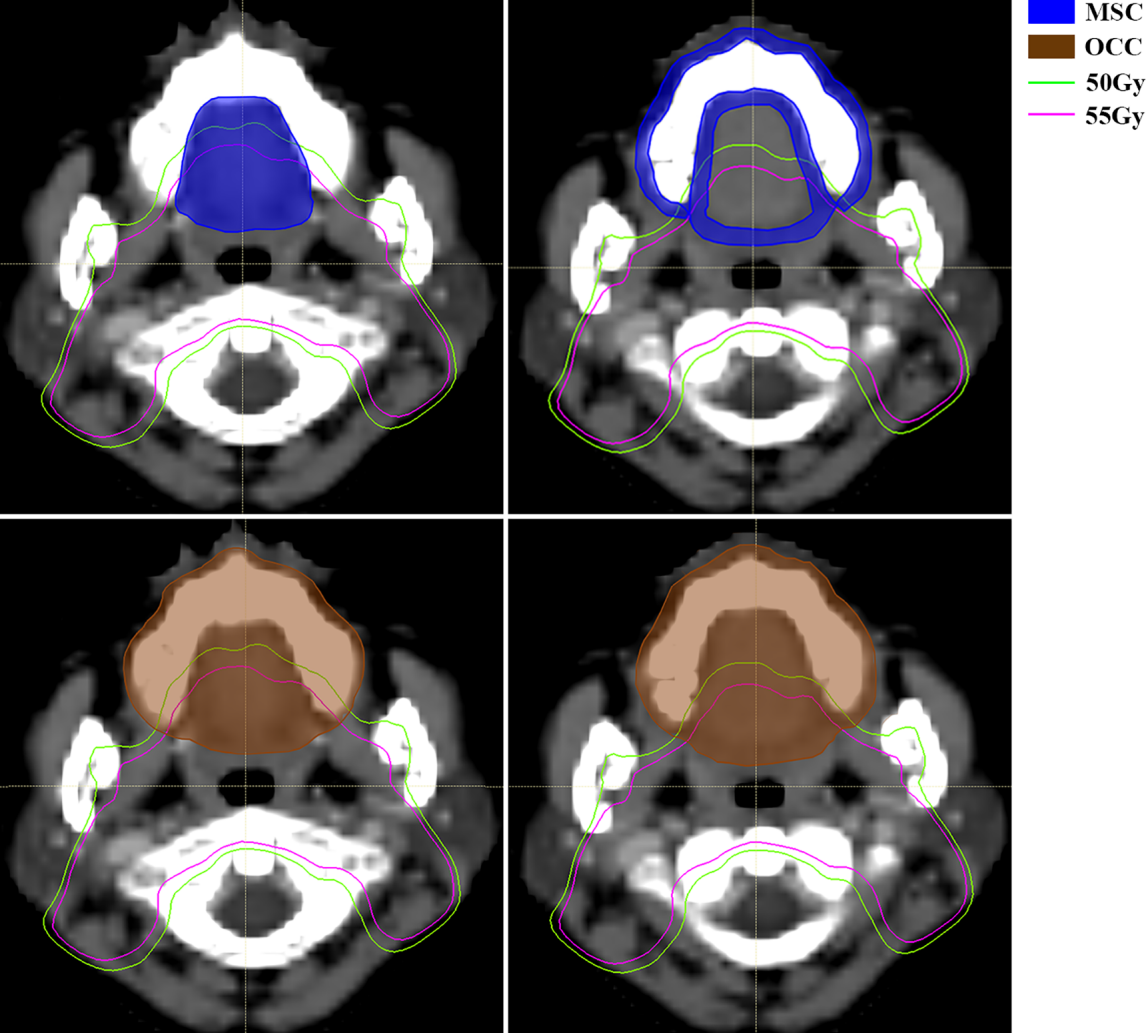
Computed tomography (CT) scan of a nasopharyngeal carcinoma patient with mucosal surface contours (MSC) (up, area filled with blue) and oral cavity contours (OCC) (down, area filled with brown). MSC involves the mucosal surface while OCC encompass more solid tissue, like tongue, maxillary bone, etc. The green line and pink line are isodose curve of 50Gy and 55Gy respectively. Abbreviations: OCC, oral cavity contouring; MSC, mucosa surface contouring.

For RT technique, it seems a little bit conflicted that the more advanced radiation technique increases the risk of severe oral mucositis. Further analysis reveals that median value of low dose-volume percentage in patients receiving TOMO is significantly higher than those in traditional IMRT. This is consistent with the characteristics of a wide range of low dose in tomography helical radiotherapy. Musha et al. reported that not only the high-dose anatomical region, but also the extensive low-dose region was associated with the development of mucositis (28). Hence, reduction of the low-dose volume is as important as high-dose volume in preventing oral mucositis. As this is a prospective observational study, dose limitation of OCC and MSC was not applied. According to opinion from physicist in our hospital, V10%-V20% could be lower in TOMO treatment plan if a certain dose restriction is applied. In OCC based model, it’s necessary to limit two parameters, a low dose-volume and a high dose volume, at the same time to achieve better control of severe oral mucositis during radiation. This is because the power of OCC V15% and OCC V60% alone are not effective enough to discriminate high risk of severe oral mucositis (AUC is 0.582 and 0.625 respectively). While, for MSC based model, only one dose parameter, MSC V55%, is needed to be limited. This further reflects that MSC is a more accurate delineation for oral mucosa. **Figure 4** shows the diffidence between MSC method and OCC method in computed tomography scan of a nasopharyngeal carcinoma patients in two transverse slices. Then, for the use of TOMO, if dosimetry indicates that it can increase treatment ratio in terms of organs at risk, we still recommend it. But strict dose limitation should be imposed on OCC and MSC. Whether RT technique is still a risk factor for severe oral mucositis after rigorously dose limitation warrants further study in the future.

Then, an interesting finding in this study is that overweight before radiation according to BMI classification is a risk factor for severe oral mucositis. Previous studies presented patients with weight loss exceed 5% during radiation were more likely to developed severe oral mucositis (9, 18). However, we are not sure whether malnutrition increases the risk of oral mucositis, or oral mucositis causes malnutrition, or they are just a vicious circle. In this study, we found overweight patients were more likely to develop into severe oral mucositis. This might be the characteristic of patients receiving head and neck radiation. Obese patients with a large body mass at the beginning of radiation are prone to lose weight during treatment. From this point of view, it is consistent with results of previous researches. Centripetal retraction due to weight loss results in the displacement of the target area, more normal tissue including oral mucosa is covered within the target area. In a study by Lee et al. (29), evaluation at the time of pre-radiation and mid-radiation shows tumor volume significantly reduces in 42% (67/159) NPC patients during radiotherapy. In another retrospective study by Wu (30), 33 patients with stage II–IV NPC was performed re-planning due to tumor/metastatic neck lymph node shrinkage or weight loss or both, then they were compared with 66 matched patients treated with a single IMRT plan. There was significant mean volume reduction in the gross tumor volume (GTV) of lymph nodes and primary tumor at the second per-treatment scans. Three-year local relapse-free survival was significantly higher in patients with T3 disease treated with re-planned radiation versus non-re-planned (P = 0.03), and there was improvement in the rates of mucosal toxicity (P =0.05) and xerostomia (P = 0.04) in IMRT re-planning group. Thus, re-planning radiotherapy in obese patients with significant weight loss might be of great value in reducing risk of severe oral mucositis and other toxicities. Moreover, researchers believed that obesity had a significant correlation with chronic low-grade inflammation. Inflammatory program is activated early in adipose expansion and during chronic obesity, permanently skew the immune system to a proinflammatory phenotype (31). Thus, overweight might be a heterogeneous factor, making it easy to irritate or aggravate by radiation, chemotherapy, etc. The underlying mechanism is still unknown.

RLN area irradiation is an important risk factor by using both contouring methods. In general, prophylactic coverage of the retropharyngeal lymph nodes in clinical target volume 2 (CTV2) extends from the base of skull to the caudal border of the hyoid bone or caudal border of the third cervical vertebra (C3) as the lower limit, which contains part of the posterior pharyngeal wall and adjoins the soft palate (32). The prescribed dose of planning target volume2 (PTV2 = 3mm expansion from CTV2) is from 54Gy to 58Gy as a rule, which almost always covers the posterior part of hard palate (**Figure 4** shows area covered with dose of 55Gy using pink line). This will inevitably increase the V55% and V60%. Studies demonstrated that approximately 75% of metastatic RLNs were located at the body of C1, 18% at C2 and probably less than 5% at the level of the body of C3 (33–35). Thus, in the era of precision medicine, whether CTV2 should extend to the level of hyoid bone in every patient deserves further discussion. Early division of CTV2 into two part for reducing the high dose coverage of the posterior pharyngeal wall, the soft palate and posterior part of hard palate might decrease the incidence of severe oral mucositis. However, it needs to be determined a balance between normal tissue protect and tumor control in further study.

In term of concurrent chemotherapy, a previous study deemed that a reduced accelerated repopulation was observed when it was delivered along with radiotherapy, which was significantly correlated with observed improvement in local control in head and neck cancer (36). Theoretically, such phenomenon exists in the regeneration and repair of mucosa during chemo-radiotherapy as well. However, the present study shows no increase of severe oral mucositis when concurrent chemotherapy was applied. Further subgroup analysis in patients underwent chemotherapy shows patients using cisplatin, nedaplatin and carboplatin have lower incidence of severe oral mucositis when it is compared with xeloda, tegafur and S1. It is in accordance with the main side effect observed in clinical practice, nausea and vomiting is often seen in platinum-based chemotherapy while mucositis is often seen in fluorouracil-based chemotherapy. In that way, it’s better to choose platinum-based concurrent chemotherapy if there is no contraindication.

The above two models could predict the incidence of severe oral mucositis. However, MSC based model is a briefer and more accurate method. BMI before radiation, RLN irradiation and high dose-volume percentage are features that can’t be neglected in predict severe oral mucositis. Furthermore, the present study has several limitations. Firstly, a study from the M.D. Anderson Cancer Center (37) found severe oral mucositis, even rare, could be observed after completion of radiotherapy. However, we missed the information after treatment completion since patients typically returned for follow-up examination 4 to 8 weeks after completion of treatment. Secondly, the range of dose distributions was not wide enough as the primary tumor location of included patients is NPC only. Therefore, it should be caution when applied this model to other kind of head and neck cancer patients receiving RT. Thirdly, external validation of these two models should be performed in the future.

## Conclusion

We developed two risk score models for predicting severe oral mucositis in nasopharyngeal carcinoma patients receiving chemo-radiotherapy. These models might help to discriminate high risk population in clinical practice that susceptible to severe oral mucositis and individualize treatment plan to prevent it.

## Data Availability Statement

The raw data supporting the conclusions of this article will be made available by the authors, without undue reservation.

## Ethics Statement

The studies involving human participants were reviewed and approved by Ethics Committee of Zhejiang Cancer Hospital. Written informed consent to participate in this study was provided by the participants’ legal guardian/next of kin.

## Author Contributions

Y-YC and MC provided idea of this research, P-JL and TJ designed the study and wrote the manuscript, H-ML, X-ZC, Y-YC, and MC recruited patients, S-YY and J-BF in charge of radiation plan, K-XL collected data, JZ, WS, and JC did statistical analysis. All authors contributed to the article and approved the submitted version.

## Funding

This study was funded by grant from National Nature Science Foundation of China (No. 81672971).

## Conflict of Interest

Authors WS and JC was employed by Hangzhou YITU Healthcare Technology Co. Ltd.

The remaining authors declare that the research was conducted in the absence of any commercial or financial relationships that could be construed as a potential conflict of interest.
