# Supplementary material for: Predictive Model and Precaution for Oral Mucositis During Chemo-Radiotherapy in Nasopharyngeal Carcinoma Patients
Source: Front Oncol. 2020 Nov 5;10:596822. doi: 10.3389/fonc.2020.596822 (PMC7674619; doi:10.3389/fonc.2020.596822)
Supplement: Supplementary file 1 [file Table_1.docx]

Figure S1


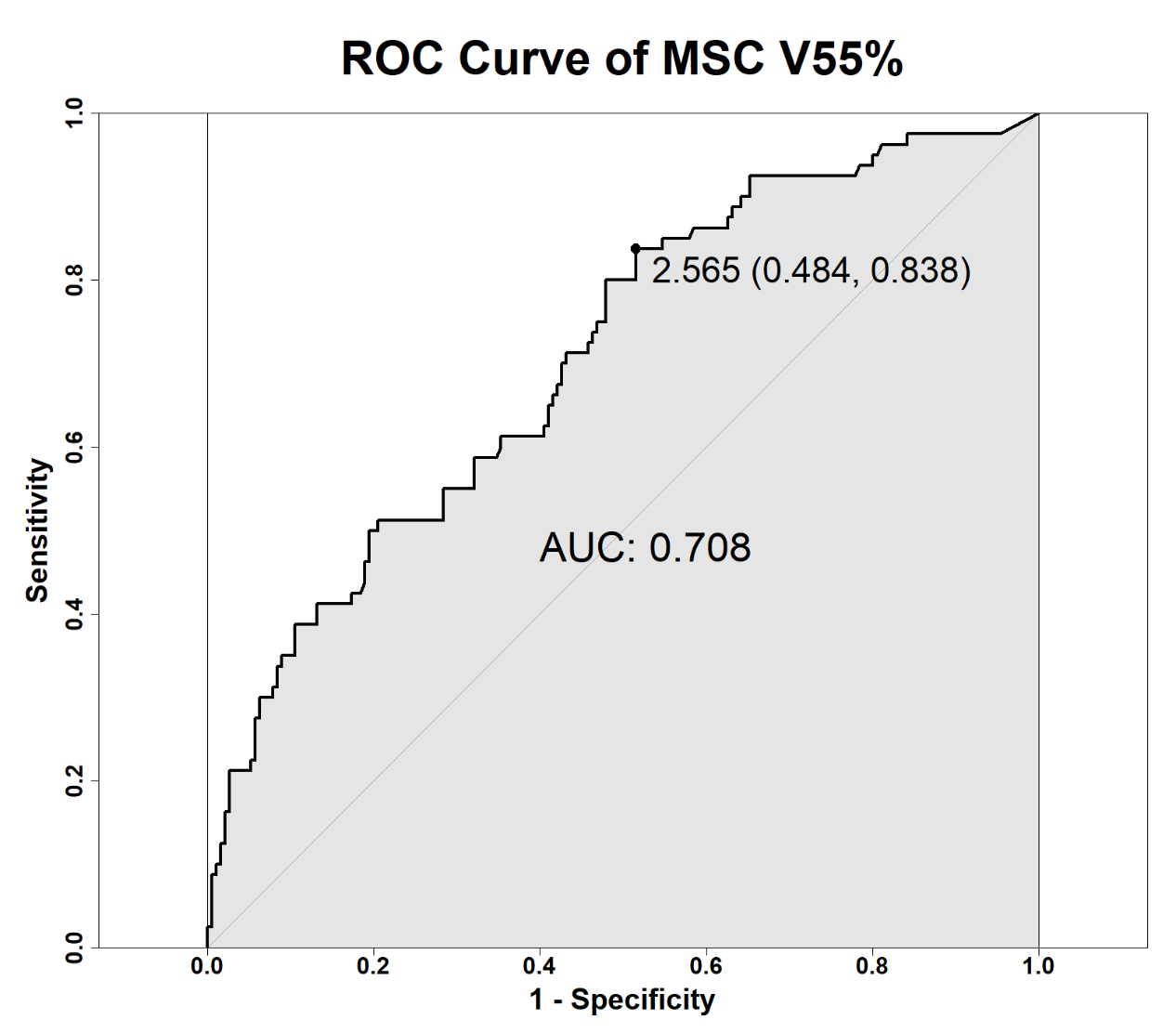


Figure S2


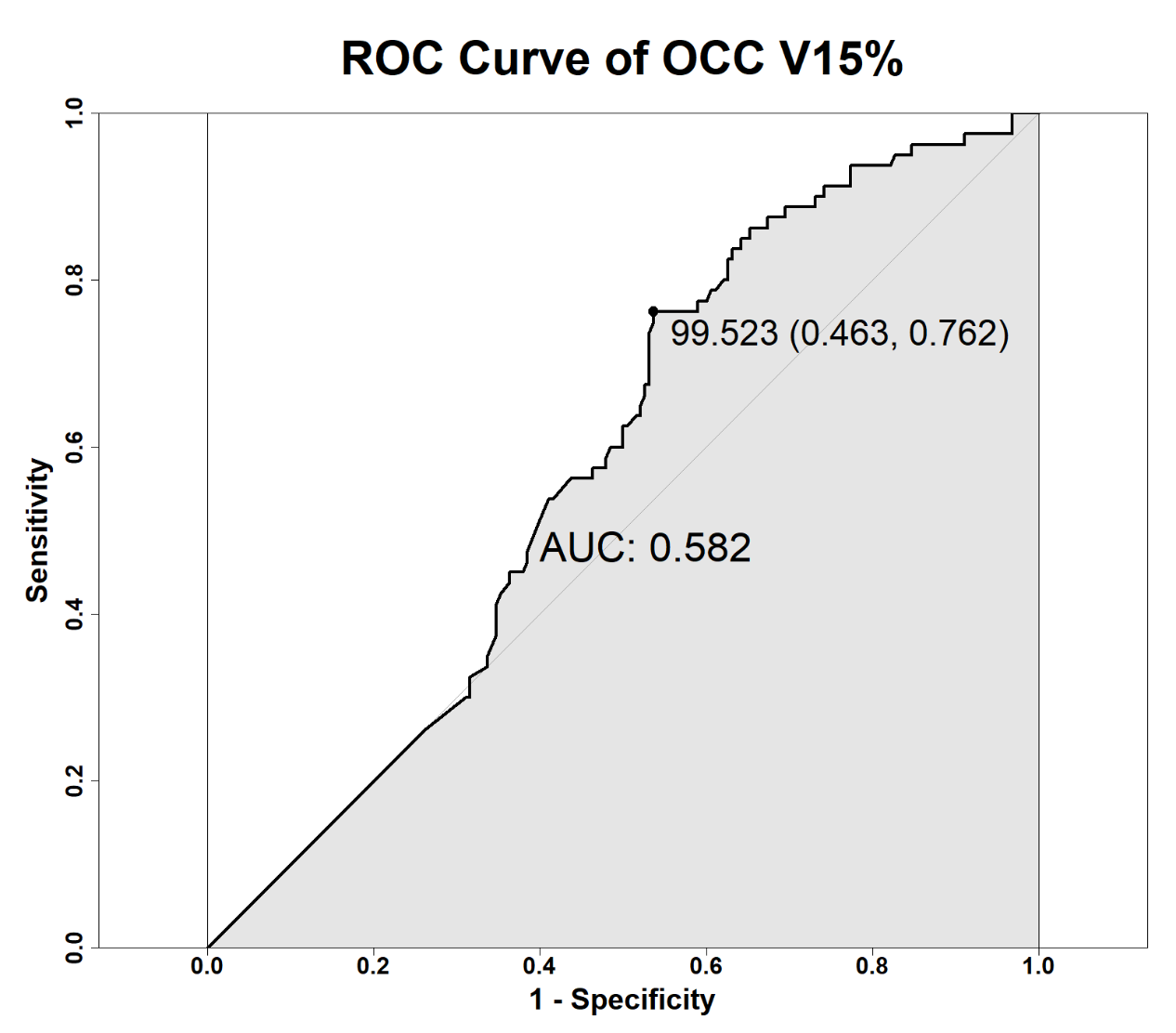


Figure S3


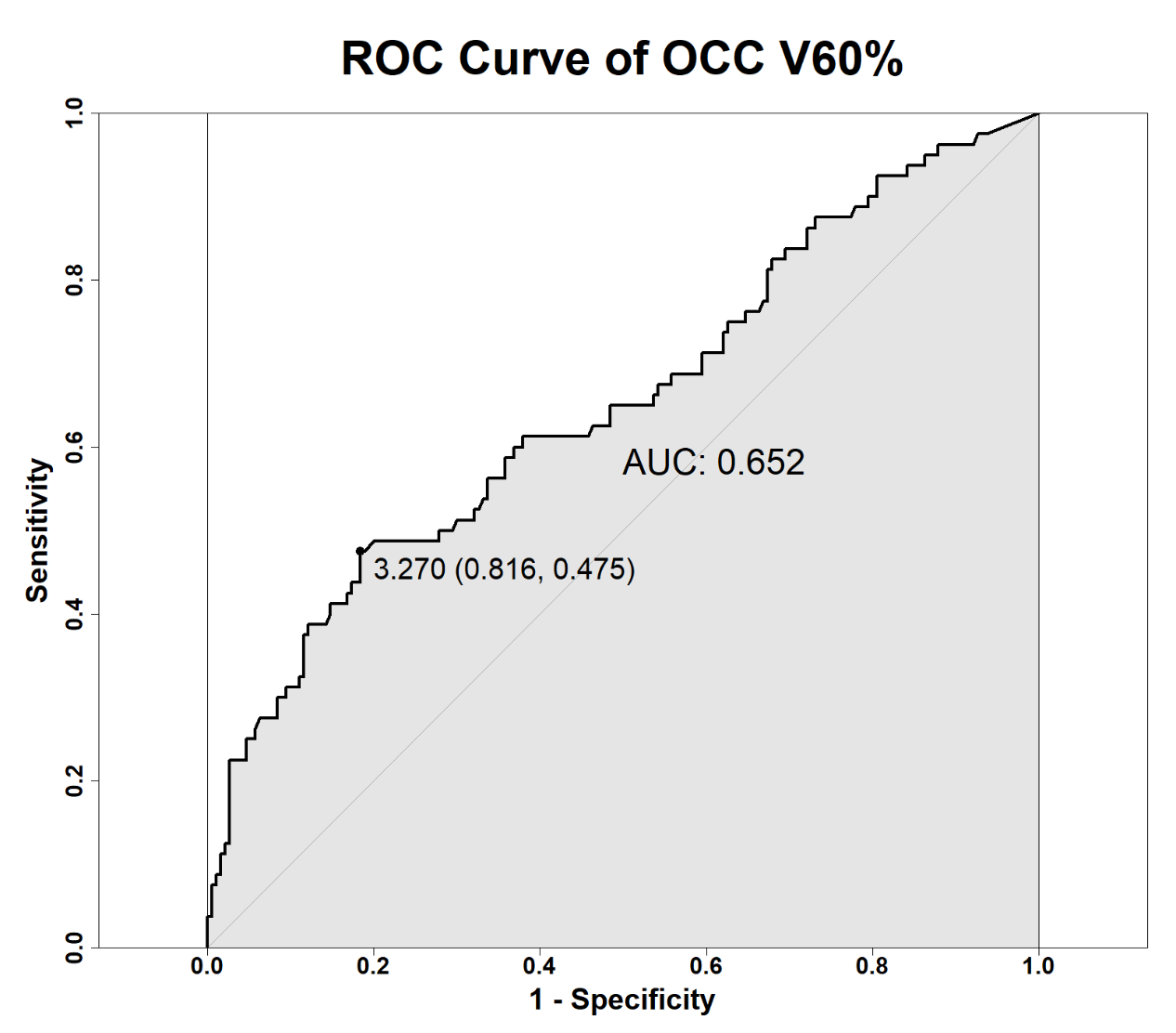


*Table S1.* *Chi-square test of mucositis in different concurrent chemotherapy agents.*

|  | Concurrent chemotherapy | | P value |
| --- | --- | --- | --- |
|  | DDP/NDP/Carboplatin | Others |  |
| Mucositis | N (%) | N (%) | 0.029^*^ |
| Grade 0-2 | 158(72.1) | 8(47.1) |  |
| Grade 3 | 61(27.9) | 9(52.9) |  |

Note: Others=capecitabine, xeloda and S1. *Statistically significant at p=0 .05 level.

*Table S2. Chi-square test of mucositis in the different group of concurrent chemotherapy or concurrent nimotuzumab*

| Mucositis | Concurrent chemotherapy | | P value |
| --- | --- | --- | --- |
|  | Concurrent nimotuzumab | None concurrent nimotuzumab |  |
|  | N (%) | N (%) |  |
| Grade 0-2 | 83 (64.3) | 83 (77.6) | 0.027^*^ |
| Grade 3 | 46 (35.7) | 24 (22.4) |  |
| Mucositis | None Concurrent chemotherapy | | P value |
|  | Concurrent nimotuzumab | None concurrent nimotuzumab |  |
|  | N (%) | N (%) |  |
| Grade 0-2 | 18 (72.0) | 6 (66.7) | 0.763 |
| Grade 3 | 7 (28.0) | 3 (33.3) |  |

Note: *Statistically significant at p=0 .05 level.

*Table S3. Comparison of dose-volume percentage in traditional IMRT and TOMO*

| Vx  (%) |  | OCC | | | | MSC | | | |
| --- | --- | --- | --- | --- | --- | --- | --- | --- | --- |
|  | RT technique | Median | Q25 | Q75 | P value | Median | Q25 | Q75 | P value |
| V5 | IMRT | 100.00 | 99.97 | 100.00 | $<$0.001^*^ | 100.00 | 99.88 | 100.00 | $<$0.001^*^ |
|  | TOMO | 100.00 | 100.00 | 100.00 |  | 100.00 | 100.00 | 100.00 |  |
| V10 | IMRT | 99.99 | 99.86 | 100.00 | $<$0.001^*^ | 99.02 | 96.90 | 99.93 | $<$0.001^*^ |
|  | TOMO | 100.00 | 100.00 | 100.00 |  | 100.00 | 100.00 | 100.00 |  |
| V15 | IMRT | 99.43 | 97.71 | 99.96 | $<$0.001^*^ | 93.54 | 89.10 | 95.85 | $<$0.001^*^ |
|  | TOMO | 99.93 | 99.26 | 100.00 |  | 99.69 | 97.85 | 100.00 |  |
| V20 | IMRT | 95.20 | 91.77 | 97.71 | $<$0.001^*^ | 83.24 | 78.56 | 87.17 | 0.002^*^ |
|  | TOMO | 91.21 | 83.62 | 96.09 |  | 86.54 | 80.43 | 92.58 |  |
| V25 | IMRT | 85.44 | 80.36 | 90.70 | $<$0.001^*^ | 70.12 | 64.54 | 76.59 | $<$0.001^*^ |
|  | TOMO | 68.62 | 60.51 | 76.38 |  | 64.75 | 58.47 | 72.00 |  |
| V30 | IMRT | 72.04 | 65.19 | 78.00 | $<$0.001^*^ | 55.16 | 49.16 | 62.25 | $<$0.001^*^ |
|  | TOMO | 45.31 | 37.82 | 54.69 |  | 48.58 | 42.98 | 53.76 |  |
| V35 | IMRT | 52.02 | 43.59 | 60.32 | $<$0.001^*^ | 39.41 | 34.26 | 46.52 | 0.008^*^ |
|  | TOMO | 29.43 | 21.56 | 35.38 |  | 36.17 | 31.42 | 42.63 |  |
| V40 | IMRT | 30.98 | 23.58 | 38.03 | $<$0.001^*^ | 28.74 | 22.36 | 33.94 | 0.700 |
|  | TOMO | 18.58 | 12.74 | 24.63 |  | 27.19 | 21.95 | 33.53 |  |
| V45 | IMRT | 18.52 | 14.34 | 23.58 | $<$0.001^*^ | 18.02 | 11.60 | 23.66 | 0.047^*^ |
|  | TOMO | 11.49 | 7.37 | 17.12 |  | 19.29 | 14.01 | 25.57 |  |
| V50 | IMRT | 11.02 | 8.14 | 14.78 | $<$0.001^*^ | 7.94 | 3.51 | 12.14 | $<$0.001^*^ |
|  | TOMO | 6.56 | 3.81 | 10.61 |  | 12.06 | 7.27 | 18.37 |  |
| V55 | IMRT | 5.51 | 3.71 | 8.17 | $<$0.001^*^ | 2.00 | 0.35 | 4.53 | $<$0.001^*^ |
|  | TOMO | 3.16 | 1.56 | 6.41 |  | 5.62 | 2.23 | 11.56 |  |
| V60 | IMRT | 2.57 | 1.29 | 3.86 | $<$0.001^*^ | 0.09 | 0.00 | 0.83 | $<$0.001^*^ |
|  | TOMO | 0.95 | 0.24 | 3.01 |  | 1.31 | 0.21 | 4.88 |  |
| V65 | IMRT | 0.72 | 0.21 | 1.60 | $<$0.001^*^ | 0.00 | 0.00 | 0.03 | $<$0.001^*^ |
|  | TOMO | 0.12 | 0.00 | 0.97 |  | 0.09 | 0.00 | 1.28 |  |
| V70 | IMRT | 0.05 | 0.00 | 0.34 | $<$0.001^*^ | 0.00 | 0.00 | 0.00 | $<$0.001^*^ |
|  | TOMO | 0.00 | 0.00 | 0.09 |  | 0.00 | 0.00 | 0.09 |  |

Abbreviation: MSC=mucosa surface contour, OCC=oral cavity contour, Q=quartile, Vx= volume of region covered with ≥x Gy, RT=radiation, IMRT= traditional intensity-modulated radiation therapy, TOMO=helical tomography radiotherapy. Note: *Statistically significant at p=0 .05 level.
